# Influence of tau on non‐traditional memory scores in early‐onset Alzheimer’s disease

**DOI:** 10.1002/alz.095765

**Published:** 2025-01-09

**Authors:** Ralitsa V. Kostadinova, Justin Bushnell, Dustin B. Hammers, Liana G. Apostolova, David G. Clark

**Affiliations:** ^1^ Indiana University School of Medicine, Indianapolis, IN USA; ^2^ Department of Neurology, Indiana University School of Medicine, Indianapolis, IN USA; ^3^ Indiana Alzheimer’s Disease Research Center, Indianapolis, IN USA

## Abstract

**Background:**

A common neuropsychological test for assessing episodic memory is the Rey Auditory Verbal Learning Test (RAVLT), a sequence of 8 word‐list learning and recall tasks (five learning trials, immediate recall of an intrusion list, short‐delay and long‐delay recall). There is extensive research correlating patterns of RAVLT performance with clinical dementia syndromes, but little work relating these patterns to biomarkers in early‐onset dementia. Here, we analyze the relationship between patterns of tau deposition and RAVLT performance in early‐onset populations.

**Method:**

We transcribed RAVLT recordings from 249 subjects in the Longitudinal Early‐Onset Alzheimer’s Disease Study (LEADS). We calculated three composite scores from scores on the individual RAVLT tasks: learning ratio, raw learning score, and recency ratio. We then performed principle components analysis (PCA) on tau measurements in 108 regions of interest, identifying five components accounting for 90.9% of the variance. We entered RAVLT composite scores as dependent variables in a series of linear regression models. The PCA components, along with diagnostic syndrome and nuisance variables (age, sex, education), were entered as independent variables.

**Result:**

Principal component 1 loaded positively in all ROIs in both hemispheres, with weaker loadings in the motor strip, occipital region, and subcortical nuclei. Principal component 5 loaded positively on left > right temporal lobes and white matter. These two were significant predictors of both learning ratio and raw learning score, showing that an increase in tau affects the performance of these RAVLT metrics. Loadings for principal component 4 were more complex, but in general were positive in the right > left hemisphere, including parietal lobes and superior temporal gyri, with negative loadings elsewhere in the temporal lobes. This component was a significant predictor of recency ratio. In all cases, regression coefficients were negative, indicating that tau within ROIs with positive loadings was negatively correlated with the memory score in question.

**Conclusion:**

RAVLT measures are sensitive to the effects of tau deposition in early‐onset Alzheimer’s disease. Further work is needed to evaluate these scores as predictors of specific forms of pathology in early‐onset dementia.

